# Is it time for insect researchers to consider their subjects’ welfare?

**DOI:** 10.1371/journal.pbio.3002138

**Published:** 2023-06-01

**Authors:** Andrew Crump, Matilda Gibbons, Meghan Barrett, Jonathan Birch, Lars Chittka

**Affiliations:** 1 Centre for Philosophy of Natural and Social Science, London School of Economics and Political Science, London, United Kingdom; 2 Animal Welfare Science and Ethics, Royal Veterinary College, North Mymms, Hertfordshire, United Kingdom; 3 School of Biological and Behavioural Sciences, Queen Mary University of London, London, United Kingdom; 4 Department of Biology, California State University Dominguez Hills, Carson, California, United States of America

## Abstract

Recent evidence suggests that at least some insect species might plausibly feel pain. This Perspective argues that researchers need to rethink the welfare implications of these findings for experiments that use insects.

## Teaser

Recent evidence suggests that at least some insect species might plausibly feel pain. These findings should prompt researchers to think about the welfare implications of insect experiments.

Do insects feel pain? Pain is an unpleasant subjective experience that often accompanies injury. This feeling is distinct from nociception: the unconscious process of detecting and avoiding harmful stimuli. Pain is hard to identify in nonhuman animals because unpleasant feelings might not accompany simple withdrawal and other behavioral responses. While we can determine that humans feel pain when they describe their experiences, this approach is obviously impossible for animals.

To understand whether an animal feels pain, researchers search for clues in their nervous systems and behavior. These clues can shift the probability of pain, even without delivering decisive proof. For example, imagine an injured dog with neuroanatomy and behavior similar to the human case. The dog winces, limps, licks their wound, avoids the place where they were injured, and seeks out analgesic drugs. None of these lines of evidence formally prove that the dog experiences pain, but collectively, they make this highly likely. Common sense dictates that such probability-shifting evidence is sufficient for us to consider the dog’s welfare.

What, then, can we say about the probability of pain in insects—and the potential importance of insect welfare? Some scientists have expressed skepticism about insect pain, pointing to their small nervous systems and apparently reflexive nocifensive behaviors [1]. Nonetheless, recent work has undermined these assertions (e.g., [2,3]).

In a comprehensive review, we evaluated over 300 studies to understand the current state of evidence relevant to pain in 6 major insect groups [3]. We adopted a contemporary framework for assessing evidence relevant to pain, which has previously guided welfare policy [4]. The framework includes 8 criteria, encompassing both whether the animal’s nervous system might support pain and whether their behavior likely involves centralized, integrative processing with the sensory and affective aspects of pain (Box 1). Each criterion adds to the case for pain: the more criteria fulfilled, the higher the likelihood.

**Box 1. Eight criteria for assessing evidence relevant to pain in animals [4]**

1. **Nociceptors.** The animal possesses receptors sensitive to noxious stimuli (nociceptors).
2. **Integrative brain regions.** The animal possesses integrative brain regions capable of integrating information from different sensory sources.
3. **Integrated nociception.** The animal possesses neural pathways connecting the nociceptors to the integrative brain regions.
4. **Analgesia.** The animal’s behavioral response to a noxious stimulus is modulated by chemical compounds affecting the nervous system in either or both of the following ways:
  - The animal possesses an endogenous neurotransmitter system that modulates (in a way consistent with the experience of pain, distress, or harm) their responses to threatened or actual noxious stimuli.
  - Putative local anesthetics, analgesics (such as opioids), anxiolytics, or antidepressants modify an animal’s responses to threatened or actually noxious stimuli in a way consistent with the hypothesis that these compounds attenuate the experience of pain, distress, or harm.
5. **Motivational trade-offs.** The animal shows motivational trade-offs, in which the disvalue of a noxious or threatening stimulus is weighed (traded-off) against the value of an opportunity for reward, leading to flexible decision-making. Enough flexibility must be shown to indicate centralized, integrative processing of information involving an evaluative common currency.
6. **Flexible self-protection.** The animal shows flexible self-protective behavior (e.g., wound tending, guarding, grooming, rubbing) of a type likely to involve representing the bodily location of a noxious stimulus.
7. **Associative learning.** The animal shows forms of associative learning in which noxious stimuli become associated with neutral stimuli, or in which novel ways of avoiding noxious stimuli are learned through reinforcement. These forms of associative learning go beyond classical conditioning in which a single conditioned stimulus overlaps temporally with an unconditioned stimulus.
8. **Analgesia preference.** The animal shows that they value a putative analgesic or anesthetic when injured in one or more of the following ways:
  - The animal learns to self-administer putative analgesics or anesthetics when injured.
  - The animal learns to prefer, when injured, a location at which analgesics or anesthetics can be accessed.
  - The animal prioritizes obtaining these compounds over other needs (such as food) when injured.

What is the neural evidence relevant to insect pain? Briefly, insects have nociceptors that respond to mechanically, chemically, and thermally noxious stimuli. In adult insects that have been studied, these nociceptors connect to higher-order brain regions that integrate nociceptive and other sensory information (important for generating a unified stream of experience). Local analgesics also affect insect nervous systems, modifying behavior in ways consistent with the hypothesis that insects feel pain. However, these analgesics also influence locomotion and learning, which could be potential confounds. There is better evidence for anxiolytic and antidepressant effects. For example, the vertebrate anxiolytic γ-oryzanol reduces “anxiety-like” light avoidance in fruit flies. This is not direct evidence for pain but may indicate some form of subjective experience.

Behavioral studies also suggest that insect pain is plausible. Tobacco hornworm larvae appear to flexibly tend the precise site of an injured proleg, undermining claims that insects do not tend their wounds (e.g., [1]). Moreover, bumblebees can trade-off competing motivations, enduring aversive heat to feed on sugar water [2]. This trade-off relied on memories of rewarding and aversive stimuli, as bees made similar choices in tests where neither sugar nor heat were present (only conditioned stimuli). Such flexible behavior tells us that heat avoidance is not simply a nociceptive reflex. It points to a mental “common currency,” which represents different options’ value and disvalue and enables comparisons. In humans, feelings of pleasure and pain plausibly perform that function.

What is the evidence against insect pain? In one study, injured honeybees did not self-administer an analgesic (morphine) more than uninjured bees [5]. This is unsurprising since insects lack opioid receptors to detect morphine [3]. However, using opioid receptors as evidence relevant to pain is too vertebrate centric. Further, the morphine and standard feeders were presented simultaneously. In this setup, even an animal with opioid receptors might not selectively associate the morphine feeder with morphine’s later effects.

Another line of negative evidence comes from some insects’ unresponsiveness to injury. For example, Eisemann and colleagues anecdotally reported that an insect with an injured tarsus continued using the leg with equivalent force [1] (though force was not measured). However, such cases could reflect a suppression of the normal injury response. This happens in humans, such as soldiers who continue fighting after sustaining life-threatening injuries [6]. Indeed, insects are capable of nociception, so they can detect and respond to injury in some circumstances [3]. While observations of insects’ unresponsiveness to injury warrant further research, they ultimately cannot rule out insect pain, particularly in other contexts or in response to different noxious stimuli.

In our review, we evaluated all available evidence, including the studies noted above and many others [3]. Given the weak negative evidence and some positive evidence, we concluded that several insect groups may plausibly feel pain. The strongest evidence (6 of 8 criteria fulfilled) came from adult flies and cockroaches, which both include well-studied model species. Adult bees, wasps, and ants satisfied 4 criteria, which this framework describes as substantial evidence for pain. Importantly, we found no cases of an adult insect being tested for a criterion and convincingly failing. However, there were considerable evidence gaps, since pain-relevant neurobiology and behavior have never been studied in most insects. Absence of evidence, though, is not evidence of absence.

Insect researchers are rarely asked to think about their subjects’ welfare. However, most people agree that humans should, where feasible, at least consider the welfare of animals who plausibly feel pain. The perception of ethical practices also determines public trust in science, including invertebrate research [7,8]. The scientific community might, thus, choose to lead in thinking about insect welfare.

In our view, the “3Rs” framework [9] may be a useful starting point for entomologists interested in considering insect welfare in research and teaching settings (a framework already used by other entomologists [10]).

1. **Replace** live insects with alternative experimental models, whenever this does not interfere with experimental or teaching aims. For example, swapping part of student insect collections with iNaturalist photo collections could reduce welfare and conservation risks and provide vast, real-time data sets. We also advise against blanket recommendations to “replace” vertebrates with insects.
2. **Reduce** the number of insects used in each study, testing only the minimum necessary to detect an effect. This could minimize both potential welfare impacts and unnecessary labor, thereby increasing research productivity and cost effectiveness.
3. **Refine** insect experiments. To avoid unnecessary harm, possibilities include standardizing rearing conditions according to best practice, introducing analgesics or anesthetics for invasive procedures, and humanely killing insects. These approaches could improve both insect welfare and scientific reproducibility.

Our aim is to find win–win cases for both insect welfare and research or teaching aims (e.g., [10]). Notably, even skeptics of insect pain have endorsed some of these suggestions as precautionary measures (e.g., [1]), and the 3Rs are already widely used in vertebrate research.

The scientific community’s most impactful change would be to study insect neurobiology and behavior relevant to pain. Major insect groups (such as beetles) and important pain indicators (such as analgesic self-administration) have barely been investigated. While there is no definitive proof of pain in insects, we think there is enough evidence for the hypothesis to warrant greater scientific attention.
